# Patterns of paternity: insights into mating competition and gene flow in a recovering population of humpback whales

**DOI:** 10.1098/rsos.241424

**Published:** 2025-01-08

**Authors:** Franca Eichenberger, Emma L. Carroll, Claire Garrigue, Debbie J. Steel, Claire D. Bonneville, Luke Rendell, Ellen C. Garland

**Affiliations:** ^1^ Sea Mammal Research Unit, Scottish Oceans Institute, School of Biology, University of St Andrews, St Andrews, Fife KY16 8LB, UK; ^2^ School of Biological Sciences, University of Auckland – Waipapa Taumata Rau, Auckland, New Zealand; ^3^ UMR ENTROPIE (IRD, Université de La Réunion, Université de la Nouvelle-Calédonie, IFREMER, CNRS, Laboratoire d’Exellence – CORAIL), Nouméa 98848, New Caledonia; ^4^ Opération Cétacés, Nouméa 98802, New Caledonia; ^5^ Marine Mammal Institute, Oregon State University, Newport, OR 97365, USA

**Keywords:** reproductive success, sexual selection, population recovery, paternity analysis, gametic mark-recapture, humpback whale

## Abstract

Variation in reproductive success is a fundamental prerequisite for sexual selection to act upon a trait. Assessing such variation is crucial in understanding a species’ mating system and offers insights into population growth. Parentage analyses in cetaceans are rare, and the underlying forces of sexual selection acting on their mating behaviours remain poorly understood. Here, we combined 25 years of photo-identification and genetic data to assess patterns of male reproductive success and reproductive autonomy of the New Caledonian (Oceania, South Pacific) humpback whale breeding population. Paternity analysis of 177 mother–offspring pairs and 936 males revealed low variation in male reproductive success (average 1.17 offspring per father) relative to other polygynous species. The observed skew in success was higher than expected under random mating and skewed overall towards males (93%) without evidence of paternity over the study period. Finally, an updated male gametic mark-recapture abundance estimate of 2084 (95% confidence interval = 1761–2407, 1995–2019) fell between previous census estimates of the New Caledonian population and the wider Oceanian metapopulation. Our results provide critical insights into the mating competition of male humpback whales and population dynamics across Oceanian populations, two important factors affecting the slow recovery from whaling across the South Pacific region.

## 1. Introduction

Multiple, often intertwining, factors shape variation in reproductive success—a fundamental prerequisite for sexual selection to act upon a trait. Within a species' mating system, males and females follow different, yet often interdependent, reproductive strategies making sexual selection a co-evolutionary process between both sexes (e.g. female mate choice) [1,2]. Ecological, demographic and social factors influence the spatial and temporal distribution of resources and mates, and thus, focusing on the male perspective, the degree of control or monopolization a male can hold over the mating opportunities of a female [3]. The greater the control, the higher the potential for polygyny (successful males mate with multiple females), and the larger the potential for variation in reproductive success. However, this influence is bidirectional, as the variation in reproductive success among individuals within a population can reciprocally affect several social factors [4].

Variation in reproductive success directly relates to the number of reproductively successful individuals, and thus, affects the effective population size per generation (Ne) [4]. In long-lived and age-structured species with overlapping generations, it is often easier and more relevant to estimate the effective number of breeders in one reproductive cycle (Nb) [5–8]. Both quantities (Ne and Nb) are directly shaped by patterns of reproductive success in a population: variation in reproductive success and reproductive skew. For example, variation in reproductive success can lead to a high reproductive skew, meaning that certain individuals are contributing more to the gene pool of a population than others. This in turn means that fewer individuals are successfully reproducing than is expected based on the census population size (Nc). High reproductive skew lowers Ne and Nb relative to Nc, which in turn can increase the stochastic effects on the genetic structure of a population (i.e. genetic drift) and the rate of loss of genetic diversity. Combined with limited gene flow between populations, a low Ne can increase the likelihood of inbreeding in the population over time [9], and ultimately, influence its fitness and ability to adapt to environmental changes [7,8]. Understanding patterns of reproductive success (i.e. variation and skew) within a population and the connectivity between populations is therefore crucial for the conservation and sustainable management of small, exploited populations [10].

Compared with most terrestrial mammals, variation in reproductive success is often much lower in cetaceans, especially in baleen whales [11]. It is thought that this is owing to the difficulty in controlling territory in a three-dimensional underwater habitat and the often dispersed distribution of females (at least in the post-whaling era), who are typically larger than males, which lowers the potential for polygyny. However, in one species, the humpback whale (*Megaptera novaeangliae*), the male-biased sex ratio on breeding grounds [11–14] and the resulting intense competition among males, combined with their elaborate acoustic display, suggest intense sexual selection. In light of the species’ proposed polygynous mating system [13,15], these observations suggest high variation in reproductive success. Yet, the handful of previous studies on male reproductive success in humpback whales found low levels of variation in reproductive success [11,16] more similar to the findings in North Atlantic right whales (*Eubalaena glacialis*) [17] and southern right whales (*Eubalaena australis*) [18]. While the marine habitat undoubtedly explains the lower variation in male reproductive success of baleen whales in comparison to polygynous mammals on land, there are many gaps in our understanding of their reproductive behaviours and mating systems. The elusive nature and low abundance of many baleen whale species make studies on their reproduction extremely challenging, especially at the temporal scale necessary to evaluate lifetime reproductive success.

This study focuses on humpback whales on their breeding ground off the coast of New Caledonia (NC) in the South Pacific. The International Whaling Commission recognizes this breeding population as sub-stock E2 as part of the genetically and demographically distinct Oceania metapopulation (that also includes the sub-stocks of Tonga (E3), the Cook Islands (F1) and French Polynesia (F2) [19,20]). Comparisons of photo-identified whales found that movement of individuals between Oceania and the Eastern Australia population (E1) [21] and among populations within Oceania [22,23] was limited, which was further supported by statistically significant differentiation of mitochondrial DNA (mtDNA) variation among these regions [19]. The statistically significant genetic differentiation and low levels of individual movement support the assumption that Oceanian sub-stocks are demographically independent populations, that are defined as groups whose population dynamics (e.g. population growth rate) depend mainly on local birth and death rates rather than on immigration [24]. This urges the consideration of Oceanian sub-stocks as separate units of management for humpback whales in the South Pacific, and thus are referred to herein as ‘populations’.

It is important to remember that all modern research has a significantly shifted baseline in terms of population density, as historical whaling could continue to influence mating systems. Humpback whales in Oceania and throughout the Southern Hemisphere were decimated by commercial and illegal whaling deep into the twentieth century [25,26]. Many populations have since substantially recovered, some to near pre-exploitation abundance (e.g. western South Atlantic: [27]; Eastern Australia: [28]). By contrast, the abundance of the Oceania metapopulation remains low relative to presumed historical numbers [29] and has not recovered as much as a neighbouring population located in Eastern Australia [30]. Garrigue *et al*. [31] investigated the reproductive autonomy (demographic independence) of NC humpback whales in the period 1995–2001 by estimating abundance using gametic mark-recapture (GMR), which considers paternity assignments as ‘recaptures’ of previously sampled (‘captured’) males (see also [18]). They found close agreement of the gametic and census estimates, confirming the low abundance of this population and indicating its reproductive and demographic closure over the study period [31]. The movement of individuals among humpback whale breeding populations in the South Pacific affects the genetic diversity within and between populations as populations grow and recover from exploitation [32,33]. Assessing the level of gene flow between populations through paternity offers insights into the demographic independence of populations in recent generations. Such information is essential for the correct identification of management units and effective conservation of humpback whales in the Southern Hemisphere.

Here, we combined 25 years (1995–2019) of photo-identification and genetic data to assess patterns of reproductive success and reproductive autonomy of humpback whales on their breeding ground in NC. First, we conducted a paternity analysis on 177 mother–offspring pairs to assess the variation and skew in male reproductive success in comparison to expectations from sexual selection theory and random mating. We expected, based on a handful of previous paternity studies on baleen whales [17,18], including humpback whales in the Northern Hemisphere [11], a low level of variation in reproductive success and a corresponding low level of reproductive skew relative to other polygynous mammals on land. Specifically, we examined the observed male reproductive success of sampled fathers, estimated patterns of paternity in non-sampled fathers, and assessed whether males share an equal chance of siring offspring within our study population. Second, we extended a historical (1995–2001) GMR estimate [31] to investigate levels of gene flow and the reproductive autonomy of the NC breeding populations over the past two decades. Considering the low but documented interchange of males across neighbouring breeding grounds in Oceania, we expected the GMR abundance estimate to fall between previous male abundance estimates of the NC breeding population and the wider Oceanian metapopulation. Our results provide, to our knowledge, the first insights into the reproductive skew of humpback whales in the Southern Hemisphere and contribute to the wider understanding of the population dynamics across Oceania, two important factors affecting the slow recovery of humpback whales in the South Pacific, and potentially worldwide, from intensive twentieth century whaling.

## 2. Material and methods

### 2.1. Study site and data collection

**Figure 1.**
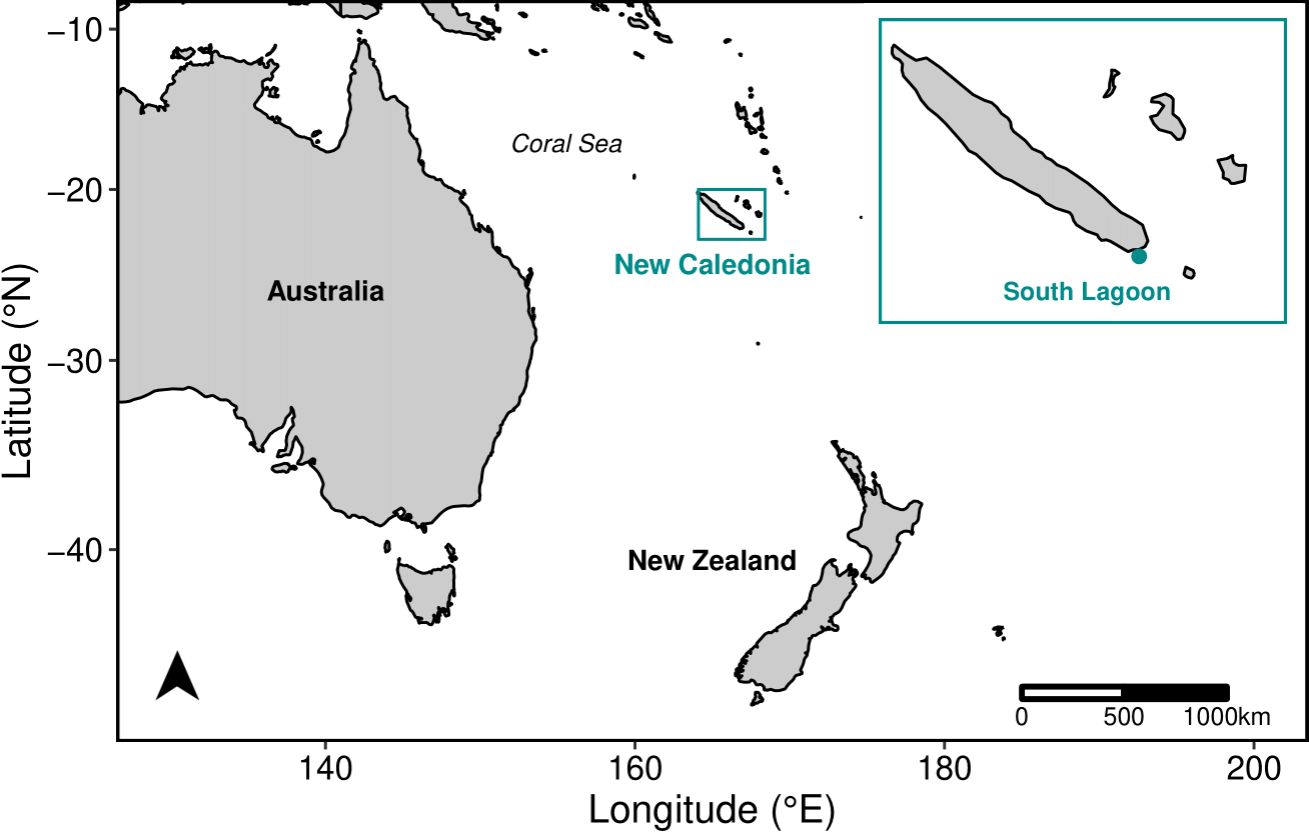
NC is located in the western South Pacific, in the Coral Sea, and is surrounded by several reefs, small islands and seamounts [36,37]. The South Lagoon at the southern end of NC is the main study area (insert).

Field surveys were conducted from small boats (6 m) in NC in the South Pacific (22°43′ S, 166°90′ E) during the humpback whale winter breeding and calving seasons (July to September) from 1995 to 2019 [34,35]. Survey effort was focused on the South Lagoon (902 days), a shallow reef area (mean depth about 50 m) located south of the mainland (figure 1) and was largely constant across the study period (electronic supplementary material, table S1). Additional surveys were conducted (264 days) along the other coasts of the mainland, around surrounding small islands (e.g. Isle of Pines, Walpole Island, Loyalty Islands), and over shallow seamounts and remote reefs all within the NC Economic Exclusive Zone ([36,37]; electronic supplementary material, supplementary methods). Survey effort at these secondary sites varied by area and year (electronic supplementary material, table S1).

During field surveys photographs and genetic samples for identification were collected [38]. Whales were carefully approached for photo-identification and to collect small skin samples using a remote biopsy system [39]. Sloughed skin was collected opportunistically from individuals engaging in surface-active behaviours [40]. Skin samples were stored in 70% ethanol at −20°C. Mother–offspring pairs were inferred based on the constant and close association of mother and calf on the breeding ground and confirmed by the presence of shared mtDNA haplotypes and Mendelian inheritance patterns in their microsatellite genotypes. Individual humpback whales were identified based on photo-identification from unique markings on the ventral surface of their tail flukes [41] and/or their genotypes [29,31]. We used photo-identification to minimize laboratory effort by genotyping identified individuals only once per season, but samples of whales that were not successfully photo-identified were analysed in every instance.

### 2.2. Genetic profiling

Genomic DNA was extracted from skin samples by either digestion with Proteinase K, and standard phenol/chloroform extraction and ethanol precipitation methods [42], as modified for small tissue samples by Baker *et al*. [43], or the Puregene DNA extraction kit (Qiagen). DNA was quantified using a NanoDrop and diluted to a concentration of 20 ng µl^−1^ with Tris-EDTA buffer.

DNA profiles for each sample were constructed using genetically identified sex, mtDNA control region haplotype and multi-locus microsatellite genotype, as previously described [29]. Samples were sexed by multiplex polymerase chain reaction (PCR) amplification of two sex chromosome-specific loci, ZFX and SRY, using the primers P1-5EZ and P2-3EZ [44] and Y53-3C and Y53-ED [45], respectively. The mtDNA haplotype of individuals was determined by Sanger sequencing an approximately 800 base-pair (bp) fragment of the 5′-end of the mtDNA control region (i.e. D-loop). PCR amplification of the mtDNA control region was carried out using the primers M13Dlp1.5 [46] and Dlp8G [47], following [19,29,31].

Multi-locus microsatellite genotypes (up to 16 loci) were constructed using previously published microsatellite loci [48–52], as described in [29] (electronic supplementary material, table S2). PCR amplification for each microsatellite locus was conducted separately, and amplified products were co-loaded in four sets of multiplex arrangements for further analysis through capillary electrophoresis on an ABI 3730xl DNA sequencer (Applied Biosystems) (see thermocycling conditions in the electronic supplementary material, table S3 and PCR set-ups in table S4). Allele scoring was performed using the GeneMapper v5 software (Applied Biosystems). Individuals typed at fewer than 12 microsatellite loci were discarded from the dataset as a measure of quality control. Genotype discrepancies between mothers and presumed calves were inspected and loci reamplified where necessary. The mean per-locus genotyping error rate for this protocol was 0.011, as reported by Constantine *et al*. [29]. Replicate genotypes were identified with the program CERVUS [53] using criteria that required exact matching for at least eight loci, supported by control region haplotypes and sex. Microsatellite allele frequencies and analysis of the probability of identity for each locus were conducted using GenAlEx 6.5 [54,55]. Population genetic tests for Hardy–Weinberg equilibrium, linkage disequilibrium and null alleles were carried out in CERVUS 3.0.7 [53] and Genepop 4.7.0 [56]. The overall (statistical) power and information content of the microsatellite markers used in this study were assessed in KinInfor, v2 [57] (electronic supplementary material, supplementary methods and table S5).

### 2.3. Paternity analysis

Using microsatellite genotypes of known mother–offspring pairs and adult males we undertook a paternity analysis over the 25 years study period by applying two different methods to assign paternities: (i) the pairwise maximum likelihood approach implemented in CERVUS v3.0.7 [53], and (ii) the full-pedigree likelihood method of Colony v2.0.6.5 [58].

In CERVUS, paternity is assigned based on the difference in the likelihood of the two most likely candidate fathers (Δ score) of each offspring [53], accounting for genotyping error. Simulations are carried out to establish the critical value of Δ determining the assignment of paternity with the specified level of statistical confidence [53,59]. We conducted simulations based on 10 000 iterations, a genotyping error rate of 0.011 [29], and required a minimum of 10 overlapping loci among mother, offspring and candidate father, with paternities assigned using strict (95%) and relaxed (80%) levels of statistical confidence (electronic supplementary material, supplementary methods and table S6). Additionally, we carried out a sensitivity analysis to assess the robustness of paternity assignments to altering values of simulation parameters (electronic supplementary material, table S7).

By contrast, Colony follows a full-pedigree likelihood approach by which parentage and sibships are inferred simultaneously, with likelihood considered over the entire pedigree configuration, rather than for pairs of individuals [58]. Considering the relationships among all genotyped individuals jointly is suggested to be a more powerful and accurate approach in paternity analysis than pairwise-likelihood methods [60]. Colony repeatedly creates different configurations by randomly allocating mother, offspring and candidate fathers into distinct family clusters over many steps, and in the end, reports the best configuration with the maximum likelihood [60] (electronic supplementary material, supplementary methods and table S8).

Paternity analyses using both methods were conducted for each year of the study period (1995–2018) to account for the growing pool of candidate fathers as calves from earlier years reached sexual maturity. The analyses were based on the yearly number of candidate males and the sampled mother–offspring pairs. Males were included in the analysis if they were of unknown age or if they were at least 5 years old, based on photo history, in the year prior to sampling the calf (i.e. year of calf conception). Although a recent update on age at sexual maturity estimates an average at 9–11 years of age based on an annual accumulation rate of earplug laminations [61], some individuals may reach sexual maturity at an earlier age [62]. Thus, to prevent the false exclusion of younger males as putative fathers, we applied an inclusive estimate of sexual maturity (5 years) for the paternity analysis. The recommended levels of statistical confidence levels for paternity analyses greatly depend on the research question of interest. More stringent criteria to avoid false inclusion increase the confidence of paternity assignments, yet at the same time will increase the risk of falsely excluding true fathers, which may result in negatively biased estimates of reproductive success [11].

Here, we followed previous baleen whale paternity studies [11,17] and created two paternity datasets based on two different confidence criteria for all further analyses on male reproductive success. The relaxed paternity dataset included paternity assignments at a confidence level of at least 80% in CERVUS while allowing for two mismatches of loci. The conservative paternity dataset included only paternity assignments at the 95% confidence level of CERVUS while allowing for zero mismatches of loci. Paternity assignments of offspring for which the two methods (CERVUS and Colony) assigned different fathers to the same offspring were excluded from the analysis. Paternity results from Colony were primarily used to confirm the assignment by CERVUS owing to the reduced power and accuracy of the full-pedigree likelihood approach of Colony resulting from small sibship sizes [60,63] in humpback whales (here, the largest known sibship size consisted of four maternal siblings; electronic supplementary material, figure S1).

### 2.4. Patterns of paternity on non-sampled fathers

To assess male reproductive skew of all sampled offspring, fathers that were not sampled need to be taken into account. We used the program DadShare (e.g. [64]) to estimate how many non-sampled males may have fathered calves. DadShare compares the genotypes of known mother–offspring pairs to infer the paternal alleles and uses these to calculate the pairwise relatedness values (*r*-values) amongst offspring following the methods of Queller & Goodnight [65]. A clustering algorithm sequentially links the most closely related individuals to form a dendrogram that is then searched for clusters of offspring that are compatible with a single father (i.e. putative siblings). Additionally, the programme performs Monte Carlo simulations to generate average *r*-values of the external nodes (putative siblings) based on different degrees of polygyny (e.g. each male siring one, two, three, four or five offspring) as a reference with which the observed average *r*-value of putative siblings can be compared. To evaluate the robustness of the simulation analysis in DadShare and the consistency of patterns of paternity on sampled and non-sampled fathers, we analysed two different subsets of sampled offspring: offspring that were assigned fathers (assigned paternities) and offspring that were left unassigned probably because their fathers were not sampled (unassigned paternities), for each of the two paternity datasets (conservative and relaxed). If the simulation analysis is robust, we would expect to find consistent results for both subsets (assigned and unassigned paternities).

### 2.5. Test of equal reproductive success

To assess whether males share equal chances of siring offspring within our study population, we compared the observed distribution of sampled males assigned as fathers of zero, one or more sampled offspring as derived from the paternity analysis to the distribution of paternities expected under the assumption of random mating. The expected distribution of paternities was generated using randomized simulations based on inputs used in the paternity analyses following methods described in Frasier *et al*. [17].

The expected paternity distribution under random mating was established using a five-step simulation process (in R Statistical Software v4.0.4; [66]): for the first year of the analyses, fathers for the number of assigned paternities in that year were randomly selected (with replacement) from the pool of mature males in that year (step 1). This process was then repeated for each year of the study period (1995–2018) (step 2). The number of offspring sired by each male was then summed across all years to generate the expected number of males assigned zero, one or more offspring if mating was random and all males had an equal probability of siring offspring (step 3). This process (steps 1–3) was repeated 1000 times to generate the mean and standard deviation (s.d.) of the number of males assigned zero, one, two, three or more offspring across all 1000 iterations (step 4). A two-sided Fisher’s exact test (FET) with *α* = 0.05 was conducted to test whether differences between the observed and expected distributions of paternities were statistically significant (step 5). Further to this, we compared the observed and the simulated mean, variance and standardized variance (SV = mean/variance; e.g. [67]) in male reproductive success for both paternity datasets (relaxed and conservative)).

### 2.6. Gametic mark-recapture to estimate abundance and assess genetic interchange

We conducted a GMR analysis in R v4.0.4 [66] to estimate the abundance of the male breeding population in NC following previously published methods (e.g. [18,31,68]). The number of assigned paternities in each of the two paternity datasets (conservative and relaxed) formed the gametic recapture of males. Chapman’s [69] modified version of the Lincoln–Peterson two-sample model was adopted for the gametic-recapture estimate equation (2.1):


(2.1) $$N_{m}=\;\frac{\left(n_{1}+1\right)\left(n_{2}+1\right)}{\left(m+1\right)}-1$$

where *N*_*m*_ is the estimated number of reproductive males, *n*_1_ is the number of mature males sampled over the entire study period (first capture), *n*_2_ is the number of offspring from sampled mother–offspring pairs (second capture), and *m* is the number of inferred paternities (recapture). The variance of the male abundance estimates (Var_*N*_) was computed as described for the Lincoln–Peterson estimator equation (2.2) and an approximate 95% confidence interval (CI) was also estimated equation (2.3):


(2.2) $$Var_{N}=\;\frac{\left(n_{1}+1\right)\left(n_{2}+1\right)\left(n_{1}-m\right)\left(n_{2}-m\right)}{{\left(m+1\right)}^{2}\left(m+2\right)}$$

(2.3) $$N_{m}\pm \;1.965\times Var_{N}^{0.5}\;$$

The GMR was conducted across a reduced study period (2000–2018). We excluded the first 5 years of the wider study period (1995–1999) from the GMR owing to the lower effort to collect data on calves before the year 2000 (electronic supplementary material, figure S2). To assess the sensitivity of our abundance estimates to different levels of confidence in the paternity assignments, we conducted the gametic-recapture analysis on both paternity datasets (conservative and relaxed), separately.

The two fundamental assumptions of GMR are that (i) the population is closed (geographically and demographically), and that (ii) all animals are equally likely to be captured in each sample [69]. During the 19-year-long GMR study period (2000–2018), the population has undergone significant input from births and deaths and is thus not closed. Moreover, both photo-ID and genetic data demonstrate some movement of individuals among Oceanian breeding grounds [22,23,70,71]. This violation of GMR’s closure assumption can bias estimates upwards as capture probabilities are reduced from the inflated number of marked animals [72]. Our assessment of reproductive skew provides insight into the validity of this assumption (ii). In this study (based on the paternity analysis), males become reproductively mature throughout the study period and therefore are eligible for ‘recapture’ as they age. Our male abundance estimates across the study period (2000–2018) might thus be best interpreted akin to a super-population abundance estimate (i.e. the total number of individuals present at the start and entering the population throughout the study period assuming no mortality) [73].

Despite the demographic independence between the NC breeding grounds and other regional breeding grounds in the South Pacific [21,22], occasional gene flow occurs among breeding grounds within Oceania and between Oceania and Eastern Australia [71]. To assess whether a substantial number of males from other populations were contributing to the paternity of NC calves, we compared our GMR abundance estimates to previous photo-ID or genetic abundance estimates of the NC breeding population and the wider Oceanian metapopulation (electronic supplementary material, table S9). Considering the low but documented migratory interchange of males across neighbouring breeding grounds in Oceania, we expect GMR abundance estimates to fall somewhere between previous male abundance estimates of the NC breeding population and the wider Oceanian metapopulation.

## 3. Results

### 3.1. Genetic profiling

The number of alleles per microsatellite locus ranged from 5 to 24 with a mean of 11.3 (s.d. = 5.6; electronic supplementary material, table S10). The 16 loci showed a mean observed heterozygosity (*H*_O_) of 0.728 (s.d. = 0.151), expected heterozygosity (*H*_E_) of 0.731 (s.d. = 0.144) and a polymorphic information content of 0.702 (s.d. = 0.159; electronic supplementary material, table S10). No linkage was detected but one locus, Ev104, deviated significantly from Hardy–Weinberg equilibrium even after Bonferroni correction and was thus excluded from all further analyses (electronic supplementary material, table S10). Using the 12 least polymorphic loci, we calculated a conservative probability of identity [74] and probability of identity among siblings [75] of 6.4 × 10^−14^ and 2.2 × 10^−05^, respectively. Considering the population size estimate of the NC population [76], the set of markers applied here offers sufficient resolution to differentiate individuals and their kin in this dataset.

Microsatellite analysis of the 2254 samples yielded 1606 unique individuals that passed the quality control for at least 12 loci typed. DNA profiles comprising genetically identified sex, mtDNA haplotype and multi-locus microsatellite genotype for all 1606 individuals were constructed. Of these, 962 were identified as male and 640 as female, while molecular sexing for the four remaining individuals failed; this resulted in a sex ratio of 1.5 : 1 males to females.

The 177 mother–offspring pairs, identified through behavioural observations and photo-ID, had genotypes consistent with Mendelian inheritance of microsatellite loci and mtDNA haplotype between parent and offspring in 172 cases. Electropherograms of mismatching loci of the five remaining mother–offspring pairs were inspected for possible genotyping errors and null alleles, and where necessary, loci were genotyped again, after which all mismatches were resolved in favour of confirming maternity.

Results of the rarefaction analysis showed that the informativeness of the five most powerful markers was sufficient to reach a confidence level of 100% to distinguish parent–offspring pairs from unrelated dyads. Using all 15 markers, parent–offspring pairs can be differentiated from half-sibling pairs in 94% and full-sibling pairs in 82% of cases. Correct distinction between half-sibling pairs and full-sibling pairs, and half-sibling pairs and unrelated dyads is expected to be achieved in 70–75% of all cases. In all five relationship distinctions, the 12 most informative markers were nearly (70–94%) as powerful as the full set of markers, thus, supporting the minimum threshold of at least 12 loci (i.e. any 12 loci from the total of 15 loci in Hardy–Weinberg equilibrium) required to accept genotypes for the paternity analysis (electronic supplementary material, figure S3, tables S11 and S12).

### 3.2. Paternity assignments

A total of 177 mother–offspring pairs were genotyped at a minimum of 12 loci and used in parentage analysis, representing 54.8% of all mother–offspring pairs observed since the start of the first survey in 1995 (*n* = 323). Of those 177 mother–offspring pairs sampled, 173 consisted of mother and calf and four consisted of mother and yearling. The pool of candidate males used in the paternity analysis consisted of 936 genotyped adult males (males of unknown age or over the age of five in at least one survey year).

Using CERVUS, 83 of 177 (47%) offspring were assigned paternities at the 80% confidence level, and 76 at the 95% confidence level from among the 936 sampled candidate males (table 1). Fewer paternities were assigned using Colony: 55 of 177 (31%) offspring were assigned a father with a posterior probability of >0.8 (table 1). For each paternity assignment (CERVUS and Colony), a minimum of 11 overlapping loci were available for comparison among offspring, mother and father (mean = 13.3 loci, max = 15 loci; electronic supplementary material, table S13). The probability of non-exclusion across all paternity assignments ranged from 3.10 × 10^−11^ to 4.27 × 10^−5^. Overall, the two methods produced similar results: 49 of 177 (27.7%) offspring were assigned the same father while 92 (51.9%) were left unassigned (all sampled fathers excluded) by both methods, 32 (18.1%) offspring were assigned a father by one or the other method, while only 4 of 177 (2.3%) offspring were assigned a different father by the two methods (electronic supplementary material, table S13). In these four disagreements, paternity assignments in Colony showed a higher number of mismatches compared with assignments in CERVUS. All paternity assignments for these four offspring were excluded from further analyses. The sensitivity analysis suggested that our paternity assignments in CERVUS were robust as the effect of shifting confidence level thresholds (i.e. critical Δ) resulting from altering input parameters in CERVUS (electronic supplementary material, figure S4) did not substantially alter the number of paternities assigned (electronic supplementary material, figure S5). Based on these results, the relaxed dataset included 79 paternity assignments of 66 fathers and the conservative dataset included 63 paternity assignments of 54 fathers (table 1; electronic supplementary material, figure S2).

**Table 1.** Paternities assigned for sampled humpback whale mother–offspring (M–O) pairs. (Included are the year the offspring were sired (year of birth – 1), the number of sampled candidate adult males (at least 5 years old in the given year or unknown age), the number of sampled M–O pairs, the number of paternities assigned in CERVUS (at the 80 and 95% confidence level) and Colony (at >0.8 posterior probability), and the number of paternity assignments in the conservative and relaxed dataset. No offspring were sampled for the year 1996 and the two study years before 1995.)

year	candidate males	M–O pairs	paternities assigned			paternity dataset	
			CERVUS (80%)	CERVUS (95%)	Colony (>0.8)	relaxed	conservative
1995	864	2	1	1	1	1	1
1996	864	0	0	0	0	0	0
1997	864	2	1	1	1	1	1
1998	864	3	1	1	1	1	1
1999	864	4	1	1	1	1	0
2000	864	7	5	4	4	5	3
2001	866	5	3	2	2	3	2
2002	866	8	2	2	1	2	1
2003	867	4	3	1	2	3	1
2004	867	6	1	1	1	1	1
2005	872	7	4	4	4	4	3
2006	874	14	10	10	5	10	7
2007	876	13	6	5	3	5	4
2008	880	5	4	4	3	4	3
2009	884	13	7	6	3	6	6
2010	889	16	7	6	6	6	5
2011	892	9	3	3	1	3	3
2012	900	8	4	4	3	4	4
2013	904	6	2	2	1	2	2
2014	906	10	6	6	4	6	5
2015	915	4	1	1	1	1	1
2016	926	13	3	3	3	3	3
2017	931	11	5	5	3	5	5
2018	936	7	3	3	1	2	1
total	936	177	83	76	55	79	63

### 3.3. Observed male reproductive success

**Figure 2.**
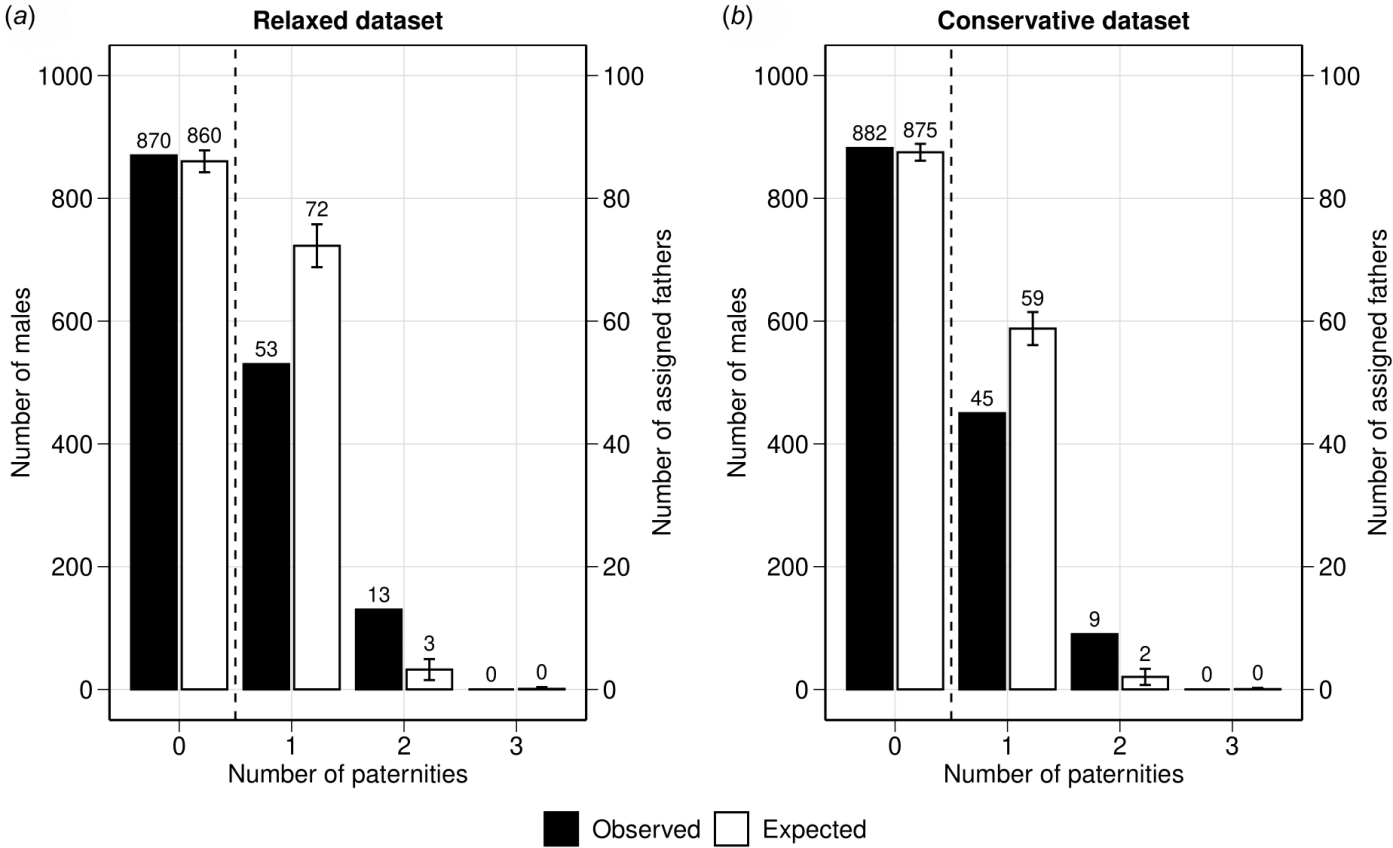
Distribution of male reproductive success in the NC humpback whale population across the entire study period (1995–2018) based on the 177 sampled mother–offspring pairs. The observed distribution (black) of reproductive success was based on the (*a*) relaxed and (*b*) conservative paternity datasets, and the mean expected distribution (white) under the assumption of equal reproductive success derived from 1000 simulations (electronic supplementary material, table S14). The left and right *y*-axes show the frequency of males that were not assigned to any of the sampled offspring and the males that were assigned one or more offspring, respectively. The dashed vertical line indicates the switch from the left to the right *y*-axis given the differences in size.

In the relaxed dataset, 870 (92.9%) males did not sire any of the 177 offspring sampled. Of males having paternity, 53 (5.7%) sired one offspring, 13 (1.4%) sired two offspring and none sired three or more offspring (figure 2*a*). A very similar distribution was seen in the conservative paternity dataset (figure 2*b*). In the relaxed paternity dataset, only one father sired twice in the same year (2006), while in the conservative paternity dataset, no father sired more than once in the same year. Both the relaxed and conservative paternity datasets produced a similar average paternity of 1.20 and 1.17 offspring/father, respectively, indicating a relatively low reproductive skew among successful males. However, the overall distribution of reproductive success across *all* males (i.e. successful and non-successful) was highly skewed towards males not siring any offspring as the majority of sampled males (93%) were not assigned as fathers to any of the sampled offspring.

### 3.4. Patterns of paternity in non-sampled fathers

**Figure 3.**
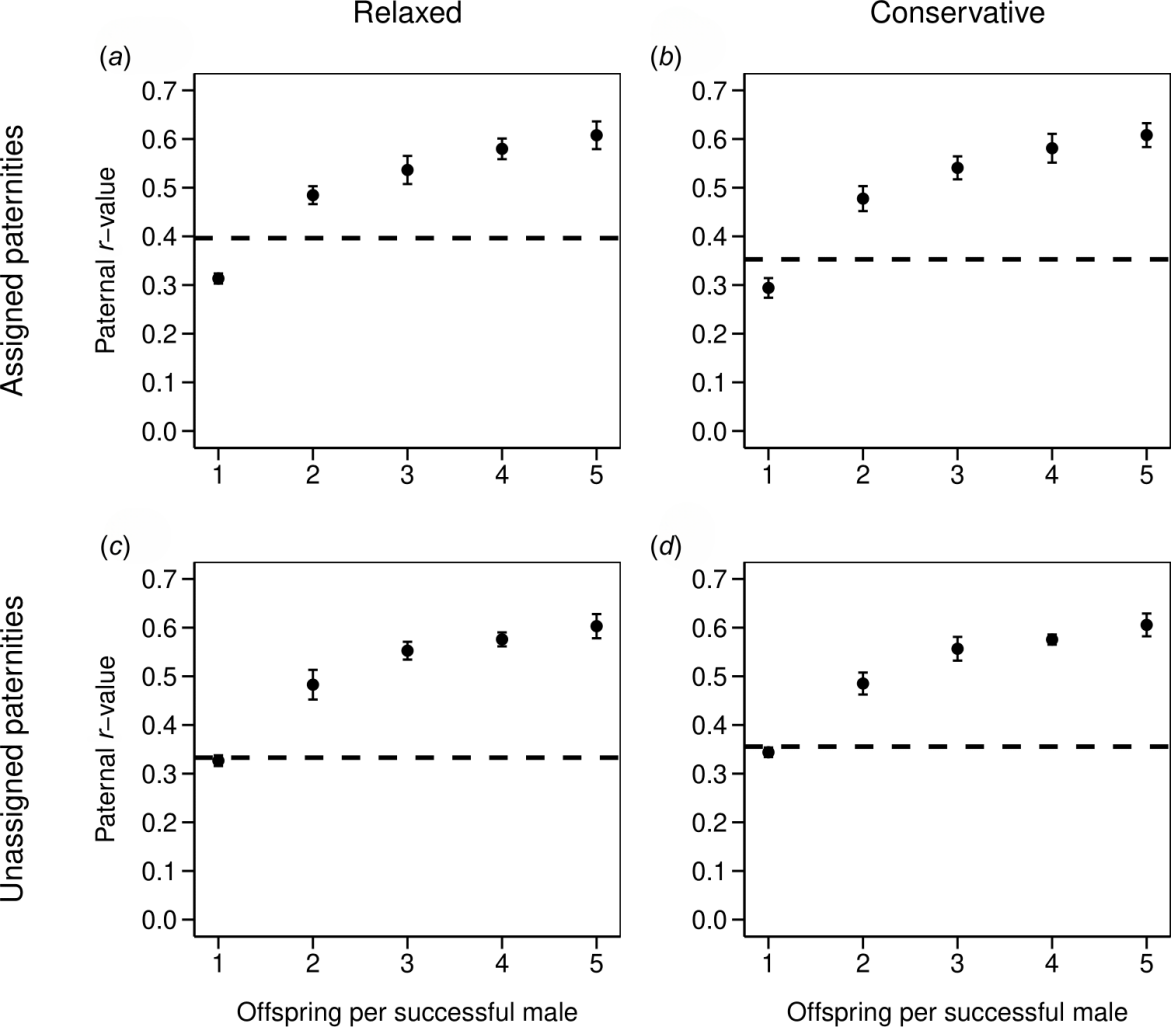
Observed paternal relatedness compared with expected values over a range of polygyny levels. Points are expected values of average paternal relatedness (*r*-values) between offspring if each successful male sired 1, 2, 3, 4 or 5 offspring. Error bars represent ±1 s.d. Observed *r*-values for each set of sampled offspring are shown as horizontal dashed lines for both paternal datasets: relaxed (*a*) and (*c*) and conservative (*b*) and (*d*). For each set of paternities, separate analyses were carried out for offspring that were assigned a father (assigned paternities, (*a*) and (*b*) and offspring whose father could not be assigned (unassigned paternities, (*c*) and (*d*)). In all cases, the observed *r*-value falls between what would be expected if each successful male sired one or two offspring.

In DadShare, the observed paternal *r*-values across offspring for which all sampled males were excluded as fathers (unassigned paternities) were 0.33 and 0.36 under relaxed and conservative criteria, respectively, and fell between the range of expected *r*-values if each successful male sired one or two offspring (figure 3). The simulation analysis in DadShare yielded consistent observed paternal *r*-values across offspring with both assigned and unassigned paternities and in both paternity datasets (assigned paternities: 0.40 and 0.35 under relaxed and conservative criteria, respectively; figure 3), as well as across all sampled offspring (0.38). Overall, these data suggest that patterns of male reproductive success are similar for sampled and non-sampled fathers in that the average number of offspring per successful male fell between one and two for sampled and non-sampled fathers.

### 3.5. Test of equal reproductive success

The expected distribution of male reproductive success derived from the random mating simulation differed significantly from the observed distribution in both the relaxed and conservative paternity datasets (FET): relaxed: *p*‐value <0.01; conservative: *p*‐value = 0.022; electronic supplementary material, table S14). This was mainly owing to fewer males than expected siring one offspring and an excess of males siring two offspring in the observed distribution compared with the expected distribution, in both datasets (figure 2). No male was expected to have sired more than two offspring based on random mating, and the sampled number of males and offspring, mirroring the observed paternity results. Although the observed variance of male reproductive success among fathers was low, it was more than 3.5 times higher than expected under random mating for the sampled pool of candidate males and offspring (relaxed: observed variance = 0.161, expected variance = 0.046; conservative: observed variance = 0.142, expected variance = 0.036; electronic supplementary material, table S15). Similarly, the observed reproductive skew among fathers was low but higher than expected under random mating, based on the average paternity (offspring/father; relaxed: observed average = 1.20, expected average = 1.04; conservative: observed average = 1.17, expected average = 1.03). These simulation results indicate that patterns of male reproductive success deviate from random mating and that certain males are more successful in siring offspring than others given the sampled number of offspring.

### 3.6. Gametic mark-recapture abundance estimate and genetic interchange

**Figure 4.**
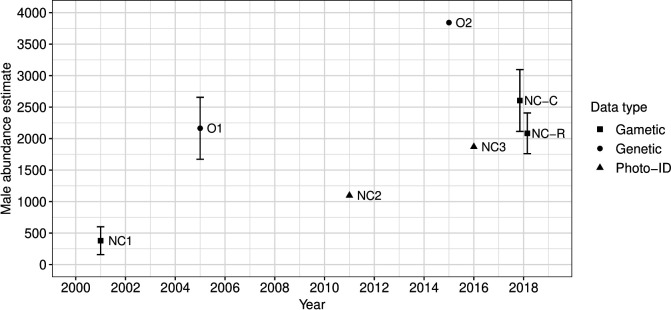
Comparison of male abundance estimates of the NC humpback whale population and Oceanian (O) metapopulation. Overall GMR estimates of the NC male breeding population derived from both paternity datasets (relaxed: NC-R; conservative: NC-C) fall between estimates of the Oceanian and the NC population. Male-specific estimates were derived from sex-unspecific estimates based on the observed male-biased sex ratio of 1.5 : 1 (*m : f*), where necessary. The horizontal position (*x*-axis) of NC-R and NC-C was jittered for illustrative purposes. O1: [29]; O2: male-specific estimate derived from [77]; NC1: [31]; NC2: male-specific estimate derived from [78]; NC3: male-specific estimated derived from [76]. Further specifications of all listed abundance estimates are in the electronic supplementary material, table S9.

There were *n*_1_ = 963 mature males, *n*_2_ = 177 offspring and *m* = 79 paternity assignments providing a GMR estimate of 2084 males (95% CI = 1761–2407) for the relaxed paternity dataset. This estimate was slightly lower than the GMR estimate of 2605 males (95% CI = 2114–3096) derived from the conservative dataset (*m* = 63 paternity assignments) (figure 4). These overall abundance estimates from the GMR across the entire study period fell between previous estimates of the NC breeding population [31,76,78] and estimates of the entire Oceanian metapopulation [29,77] (figure 4; electronic supplementary material, table S9).

## 4. Discussion

Variation in reproductive success is fodder for sexual selection, and we demonstrate here that there is a significant deviation from random mating in a recovering population of humpback whales in the South Pacific. Surprisingly, the reproductive skew is more reflective of many males not mating at all, rather than some males drastically outcompeting their rivals. While the male-biased sex ratio on the breeding ground undoubtedly contributes to the observed excess of males not being assigned any paternities, the observed non-random pattern of reproductive success further indicates that some males are more successful than others in siring offspring. Altogether, this results in fewer males successfully reproducing than is expected based on the underlying population size. This can reduce the genetic diversity of the NC population, and ultimately, its fitness and ability to adapt to environmental changes [7,8]. However, the GMR abundance estimate suggests that there are potentially higher levels of contemporary gene flow between NC humpback whales and its neighbouring breeding grounds than previously thought [31], which could reduce the negative effects of genetic drift and inbreeding on the genetic diversity of affected populations. The wide-ranging consequences of historical exploitation, combined with current ecological and demographic factors, appear to be shaping patterns of male reproductive success and population dynamics of humpback whales in the South Pacific, and potentially worldwide, today.

### 4.1. Patterns of male reproductive success

**Figure 5.**
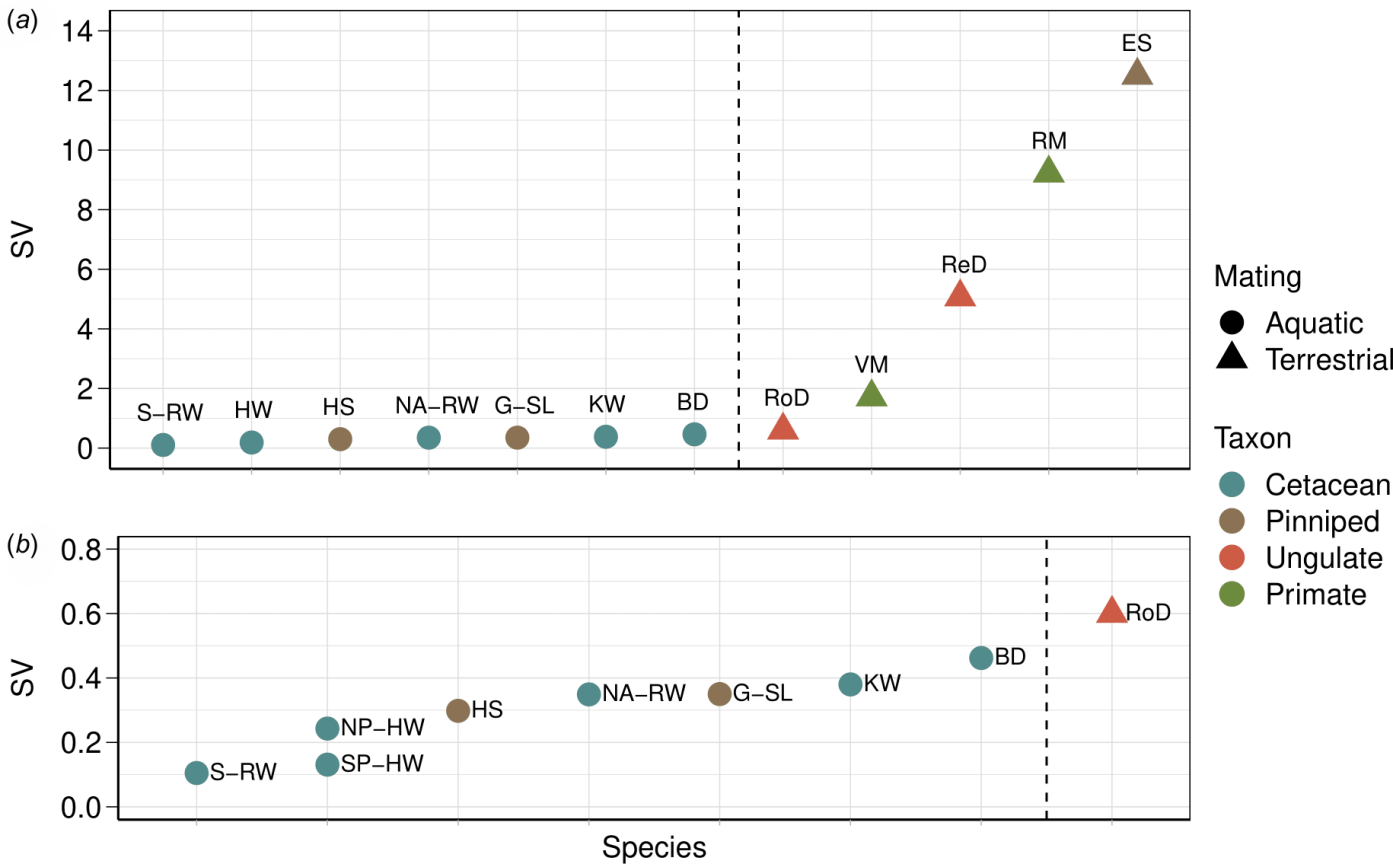
Standardized variance (SV = variance/mean) in male reproductive success (among fathers) across a range of species and taxa with (*a*) showing all species, and (*b*) a close-up of species and multiple humpback populations with SV of less than 1. This figure has been adapted and extended from [17]. Abbreviations: BD (bottlenose dolphins, *Tursiops* sp.) [79], ES (southern elephant seals, *Mirounga leonina*) [80], G-SL (Galapágos sea lion, *Zalophus wollebaeki*) [81], HS (harbour seals, *Phoca vitulina*) [82], KW (killer whale, *Orcinus orca*) [83], NA-RW (North Atlantic right whale) [17], NP-HW (North Pacific humpback whale) [11], ReD (red deer, *Cervus elaphus*) [84], RM (rhesus macaque, *Macaca mulatta*) [85], RoD (roe deer, *Capreolus capreolus*) [86], SP-HW (South Pacific humpback whale, this study), S-RW (southern right whale) [18], VM (vervet monkey, *Chlorocebus pygerythrus*) [87].

Sexual selection theory predicts high variation in reproductive success in polygynous species [3]. In the population of Southern Hemisphere humpback whales we studied, variation in male reproductive success was lower than that of other polygynous species on land, yet fell within a similar range to other aquatically mating species (figure 5), including its conspecific population in the North Pacific [11]. The synchrony of female oestrus [88], the great dispersion of females across the breeding ground, and the three-dimensional underwater habitat all reduce a male’s ability to monopolize female mating access [89]. Combined, these factors lower the degree of polygyny possible in species that mate underwater compared with terrestrially mating mammals with a polygynous mating system, and ultimately, reduce the variation in male reproductive success in cetaceans [17].

However, despite this low variation in male reproductive success, our results indicate that there was significantly more male reproductive skew than expected if mating was random. Fathers were at least 3.5 times more likely to sire more than one offspring than expected if mating was random, suggesting that some males are more successful than others in siring offspring. Such differences in reproductive success may be driven by behavioural or developmental differences among males. For example, in northern elephant seals (*Mirounga angustirostris*) male reproductive success is determined by dominance rank, which, in turn, is affected by male size [90]. A variety of male mating behaviours are frequently observed on humpback whale breeding grounds, with males physically competing to be closest to a female within competitive groups [91], escorting single females (with or without calf; [13]) and displaying elaborate songs showing high levels of complexity ([89]; electronic supplementary material, table S16). Young humpback whales may lack the skills or strength to outcompete (or ‘out-sing’) their adult competitors, yet they might engage in these behaviours to gain experience and practice their skills, as was suggested in North Atlantic right whales [92]. Female mate choice could also be playing a role in addition to male–male competition. If females prefer certain physical or behavioural traits, then males with those traits will be more successful in siring offspring than males without the trait or with a less extreme manifestation of it. A study investigating the body size of male–female dyads in humpback whales in Hawai'i using underwater videogrammetry found that mature-sized females showed a preference for larger mature-sized males [93]. This size-assortative pairing suggests that females discriminate amongst potential mates on the basis of size or age. Furthermore, the complex songs of male humpback whales have been suggested to serve as a sexual display to attract females [89,94–97], similar to birdsong [98]. In song sparrows (*Melospiza melodia*), females show a preference for males with more complex songs (e.g. larger song repertoire; [99]). The high structural complexity and constant evolution of humpback whale song [100–102], make song learning a continuous part of a male’s life. Older, more experienced males might be more skilful singers and/or better or faster learners, and thus, could be preferred by females. Despite the low variation in male reproductive success among fathers, sexual selection can still act upon song as non-random success and high skew among males provide the prerequisite for sexual selection to act upon the trait. However, a female preference for any humpback whale song characteristic (e.g. song complexity) and its possible link to male reproductive success is yet to be investigated.

Although males have been observed to engage in competitive groups, escorting and singing behaviour both across different years and within the same year [11,103], males might nevertheless favour or be more successful in one tactic compared with another depending on current conditions and opportunities. On the breeding grounds around Hawai'i, males that associated with immature-sized females tended to be either immature themselves or mature but smaller than males that associated with mature-sized females [93]. This indicates that males may adopt different reproductive tactics depending on their own body size, and that of a possible female mating partner, to avoid the costs of competing for the highest-quality females [93]. In a different study on the same breeding ground, males preferred to associate with, and competed more intensely for females with high reproductive potential (no calf vs with calf) but became progressively less choosy over the course of the breeding season as the number of females without a calf decreased [104]. Male humpback whales may thus adjust their reproductive investment or adopt a specific behavioural tactic depending on their own age, experience, body size, current condition, as well as that of their possible female mating partner or external demographic factors (e.g. number of receptive females, number of male competitors, dispersion of individuals). In male African savannah elephants (*Loxodonta africana*), energetic investment into reproduction, and ultimately, reproductive success increased with age [105–108]. Yet, despite the higher competitive ability and reproductive dominance of older males, younger males were still able to undertake opportunistic reproductive tactics that are energetically less expensive, and so still contribute to the gene pool [106,109]. Contrary to predictions from behavioural and life-history traits, the male reproductive skew of African savannah elephants was also lower compared with many mammals with a similar mating system [109]. Male humpback whales may similarly improve their reproductive chances, and ultimately their reproductive success, by adapting their reproductive investment and tactic to their current state and changing external conditions. Owing to the recent history of whaling, populations of humpback whales and other baleen whales have a larger proportion of young individuals than would be expected for a stable population [110–112]. If male reproductive tactics and reproductive success in humpback whales are age related, then commercial whaling could still have substantial effects on their behaviour and genetic structure today, mirroring the impacts of trophy hunting and ivory poaching on populations of African elephants [109].

While the marine habitat and the wide dispersion of females across the breeding ground undoubtedly reduce the variation in male reproductive success, age-related paternity success, alternative or adaptive reproductive tactics, and female mate choice can further lower it (see also [11]). Yet, low variation in male reproductive success does not necessarily imply that sexual selection acting upon male behaviour is weak. The male-biased sex ratio and the scattered distribution of females still suggest intense male competition. Male competition might be more about being able to reproduce at all rather than siring a large number of offspring. Although this does not lead to high variation in reproductive success among fathers, it still results in a highly skewed distribution of reproductive success among males. Male reproductive success in humpback whales, however, does not appear to be skewed towards few males siring a large proportion of the offspring but instead, a large proportion of males siring no offspring. Based on the estimated proportion of sampled males (approx. 50%) and mother–offspring pairs (approx. 55%) of the NC population (see §2), most sampled males (93%) showed no evidence of paternity over the 25 year study period. A paternity analysis of male humpback whale reproductive success in the North Pacific over a 6 year study period found similar results with 82% of sampled males left unassigned (40–50% estimated male population sampled; 54% of observed mother–offspring pairs sampled) [11]. Altogether, these findings suggest intense levels of competition among male humpback whales. The non-random and highly skewed distribution of reproductive success among all males shown here provides a clear foundation for sexual selection to act upon traits. This study further highlights the importance of including the number of sexually mature males that did not sire any offspring in studies while taking into account sampling issues (i.e. incomplete sampling of offspring), in contrast to assessing the variation of reproductive success of only the males that were able to reproduce. Long-term studies alone provide the opportunity to assess reproductive success on the scale of decades, allowing us to cover a considerable proportion of the lifespan of a humpback whale.

### 4.2. Gametic mark-recapture estimates and reproductive autonomy of the New Caledonian breeding population

The overall GMR male abundance estimates for the NC population fell between previous estimates of the NC breeding population and estimates of the entire Oceanian metapopulation. While this suggests that at least some males from other neighbouring Oceanian breeding grounds are contributing to the paternity of NC calves, the inferred level of gene flow varies depending on the level of confidence applied to the paternity dataset used in the GMR. The GMR estimate derived from the conservative paternity dataset lies in the centre between previous NC and Oceanian census estimates (figure 4), and therefore, suggests considerable levels of gene flow; the relaxed GMR estimate falls close to the most recent NC census estimate, reflecting its possible demographic independence and mirroring results from Garrigue *et al*. [31] over 20 years ago (see also [113]).

Previous studies on genetic differentiation between, and high song conformity within, breeding populations [19,101] in the South Pacific indicate that maternal breeding site fidelity is higher compared with the degree of interchange [114]. This reinforces the reproductive autonomy and demographic independence of the NC population. Individuals within a population show a high level of conformity to the current arrangement and content of song [96,115] while populations that are geographically closer to each other show a higher level of similarity than those further away [105]. Song transmission is suggested to occur through male movement between breeding populations, song sharing along shared or partially shared migration routes, and/or on shared summer feeding grounds [116,117]. As song evolves progressively through time and space [100,101,118], the unidirectional transmission of song eastwards across the South Pacific might too be the result of differences in population size [101]. Changes in population size might thus influence the levels of interchange between populations through the transmission of both genes and song [119].

However, there are biological reasons to consider when suggesting a higher level of interchange between the NC population and the broader Oceania metapopulation, and methodological considerations in the GMR approach that mean it is hard to draw firm conclusions. First, population growth and density-dependent gene flow could be a factor, particularly in light of the high growth rates of the neighbouring Eastern Australian population [30]. It is, however, unlikely that population growth owing to internal recruitment alone is the main factor contributing to our higher male abundance estimate for NC (figure 4). A simpler explanation, consistent with the conservative GMR estimate, is that some males from neighbouring populations contribute to the paternity of NC calves. Previous population dynamic analyses, photo-ID and genetic work [19,22,31] consistent with a degree of demographic closure were based on data collected during a time when the population was considerably smaller than it is today. More recent studies have shown a substantial number of resights [120] and longitudinal movement [121] between NC and Eastern Australia. Recovering populations of baleen whales were shown to expand their range and recolonize areas despite the typical maternal fidelity to breeding grounds [122–124]. The reported increase in abundance of the NC population [78], the Eastern Australian population [30] and the wider Oceanian metapopulation [77] might have increased and expanded the level of gene flow among humpback whale breeding populations in the South Pacific. It is also worth highlighting that mtDNA-based analyses focus on a maternally inherited DNA marker, whereas paternity analyses reflect male gene flow, and there could be sex biases in dispersal.

Additionally, caveats around GMR need to be considered. The GMR estimates derived from the conservative paternity dataset were higher overall and had a greater uncertainty when compared with estimates derived from the relaxed paternity dataset. By definition, the conservative dataset will have a higher false negative rate compared with the relaxed paternity dataset, which reduces recaptures and could inflate the GMR estimate and decrease its precision. Conversely, the relaxed paternity dataset may have false inclusions (father-offspring pairs that are not real), thereby inflating the overall recapture rate, and thus, reducing the derived GMR estimate compared with the conservative dataset (see also [113]). Although differences between the relaxed and conservative paternity datasets were small and overall led to similar conclusions regarding patterns of male reproductive success, these differences appear magnified in the GMR analysis. The artefactual process based on the methodology applied, should be considered when drawing biological conclusions from the GMR approach in this study and in others that have used similar approaches. Additionally, an assumption of GMR is that all males have equal capture probability; that is all males are equally likely to sire a calf. While we showed that sampled and unsampled males probably have the same chance of siring calves, the significant deviation from random mating suggests that not all males are equally likely to be recaptured in a paternity. Typically, heterogeneity in capture probability is thought to create a negative bias in capture-recapture abundance estimates [125] decreasing the population estimate, but it can vary depending on the circumstances and model applied (e.g. [126]).

## 5. Conclusion

Investigating reproductive success in baleen whales, especially in males, remains a challenge owing to their long lifespan and elusive nature. The 25 years population monitoring and extensive genetic sampling of the NC humpback whale breeding population offered the unique opportunity to explore patterns of male reproduction over almost one-third of a humpback whale’s lifespan. It consequently also allowed for the assessment of two important factors affecting the recovery of this endangered population: reproductive skew and demographic independence. Variance in male reproductive success of humpback whales in the South Pacific was relatively low, mirroring patterns of their conspecifics in the Northern Pacific [11]. Yet, sexual selection is suggested to be strong, as competition among male humpback whales may be more about being able to reproduce at all rather than siring a large number of offspring. Not all males shared equal chances of siring offspring, but what makes some males more successful than others and how female mate choice may shape patterns of male reproductive success remain open questions.

Based on the GMR estimates in this study, current levels of gene flow between NC with neighbouring populations in the South Pacific may be larger than estimated 20 years ago. Recent increases in the abundance of populations in Oceania [77,78] and Eastern Australia [30] could have expanded and increased levels of gene flow across breeding populations in recent years. More detailed analyses to revise previous records of individual and genetic interchange among populations in the South Pacific and their demographic independence are needed as levels of gene flow, as well as patterns of male reproductive success may have changed as populations recover from whaling. Through continued monitoring of their populations, future studies may assess the evolutionary consequences of commercial and illegal whaling on the demography, reproductive tactics and sexual selection of humpback whales.

## Data Availability

The dataset supporting this article has been uploaded as part of the electronic supplementary material (table S15) [127].
